# Establishment of the H8T-MG Meningioma Cell Line and Integrated Transcriptomics Reveal a Metabolic–Immune Signature in Diploid Transitional WHO Grade 1 Tumours

**DOI:** 10.3390/biom16050744

**Published:** 2026-05-19

**Authors:** Esther Mancheño-Maciá, Marina Leal-Clavel, Vanesa Escudero-Ortiz

**Affiliations:** 1 Department of Biomedical Sciences, School of Health Sciences, Universidad Cardenal Herrera-CEU, CEU Universities, 03204 Elche, Alicante, Spain; mleal.el@uchceu.es (M.L.-C.); vanesa.escudero@uchceu.es (V.E.-O.); 2 Department of Nursing and Physiotherapy, School of Health Sciences, Universidad Cardenal Herrera-CEU, CEU Universities, 03204 Elche, Alicante, Spain

**Keywords:** arachidonic acid metabolism, cannabinoid receptors, cytochrome P450, diploid transitional meningioma, inflammatory metabolic signature, meningioma cell line, protein–protein interaction network, transcriptomics, xenobiotic metabolism

## Abstract

Meningiomas are the most common intracranial tumours, yet the molecular programs underlying WHO grade 1 subtypes—particularly transitional diploid tumours—remain insufficiently defined, partly due to the scarcity of biologically faithful in vitro models. Here, we report the establishment of a long-term, genetically unmanipulated grade 1 meningioma cell line (H8T-MG) maintained under normoxic conditions in serum-containing, growth-factor-supplemented medium, together with a complementary long-term primary culture (H16T-MG), and provide an integrated descriptive and functional characterization of these models, combined with a subtype-restricted transcriptomic analysis of diploid transitional grade 1 tumours versus normal meninges. Both cultures preserved the dual meso-neuroectodermal identity characteristic of meningothelial cells, exhibiting stable adherent growth, preserved contact inhibition and a coherent immunocytochemical profile, expressing vimentin, α-SMA, nestin, connexin-43 and cannabinoid receptors—reported here for the first time in grade 1 meningioma cultures—highlighting cannabinoid-related pathways as potential targets for exploration. Transcriptomic analysis identified 51 differentially expressed genes, revealing a coherent inflammatory–metabolic programme characterised by downregulation of IL-17 and TNF signalling, cytokines and chemokines (IL6, CCL2, SELE, S100A8), together with reduced extracellular-matrix and cytoskeletal activity. In parallel, the enrichment of arachidonic acid metabolism, cytochrome-P450/xenobiotic pathways, retinol metabolism and oxidative/epoxygenase activity indicated a lipid/xenobiotic-oriented metabolic shift distinctive of this subtype. Protein–protein interaction analysis identified four hub genes—ASPN, SELE, ACKR1 and ABCB1—integrating ECM remodelling, endothelial–immune modulation and xenobiotic transport, reinforcing an immune-attenuated, metabolically adapted tumour landscape. Collectively, these findings provide the first integrated in vitro and transcriptomic characterisation of diploid transitional meningiomas, underscore the value of biologically stable models for early-stage meningioma research, and support the value of histological and ploidy stratification in grade 1 meningioma biology.

## 1. Introduction

Meningiomas are the most common primary intracranial tumours, representing approximately 38.3% of all central nervous system (CNS) neoplasms [1]. Although they are believed to arise from meningothelial (arachnoid cap) cells, their exact cellular origin has not been definitively demonstrated [2,3]. Despite their high prevalence, the molecular mechanisms driving meningioma initiation and progression remain incompletely understood [2]. According to the WHO classification, most meningiomas are grade 1 lesions (78–81%), whereas 15–20% are atypical (grade 2) and 1–4% are anaplastic (grade 3) [1,4].

At the chromosomal level, meningiomas display marked genetic heterogeneity. Although monosomies, deletions and trisomies affecting multiple chromosomes have been described, no single cytogenetic pattern reliably correlates with WHO tumour grade. Many tumours retain a normal karyotype, whereas hypoploidies and complex chromosomal alterations increase with tumour aggressiveness and typically involve chromosomes 22, 1, 3, 6, 9, 10, 14 and 18. Notably, such complex karyotypes may occasionally appear even in some grade 1 cases [5,6,7]. Importantly, large cytogenetic series have shown that diploid karyotypes represent a substantial and clinically relevant fraction of WHO grade 1 meningiomas. Together with isolated monosomy 22, they form the most common grade 1 cytogenetic subgroups, strongly associated with favourable long-term clinical outcomes [6,8,9,10].

Although they are the most common subtype, meningiomas at WHO grade 1 remain among the least explored at the molecular level. Their typically benign clinical behaviour has contributed to limited genomic and transcriptomic investigation compared with higher-grade tumours, leaving early biological programs incompletely understood [11].

Functional studies require biologically faithful and long-term meningioma models. However, primary human meningioma cells—particularly those derived from grade 1 tumours—are notoriously difficult to maintain in culture for extended periods [12], and most available long-term lines originate from atypical or anaplastic meningiomas. Only a small number of studies have reported long-term grade 1 models, often requiring genetic manipulation or non-standard culture conditions [13,14,15,16].

To elucidate the biological basis of meningioma development, it is essential to integrate their in vitro growth behaviour with immunocytochemical, genetic and genomic profiles. Although genomic approaches have advanced tumour biology, functional validation still requires reliable cellular systems, and both in vitro and in vivo studies depend on stable meningioma models [17,18]. However, human meningioma cells, especially those from WHO grade 1 tumours, are difficult to maintain in long-term culture: most available cell lines derive from atypical or anaplastic tumours, whereas primary grade 1 cultures often undergo senescence after only a few passages [12]. Only a handful of studies have reported long-term grade 1 meningioma cell lines [12,13,14,15], and nearly all required genetic manipulation or non-standard conditions, with the most recent model relying on hypoxia [15]. Therefore, robust, non-manipulated grade 1 models under normoxic conditions remain critically needed for molecular and translational research.

Within a systems–biology framework, functional enrichment analyses help uncover the molecular programs underlying tumorigenesis. Integrating differentially expressed genes (DEGs) into Gene Ontology (GO) and KEGG analyses highlights coordinated biological processes and signalling pathways not evident from single-gene inspection [19].

However, comparatively little attention has been given to the metabolic dimension of grade 1 meningiomas and its potential role in early tumour adaptation. In our subtype-focused analysis of diploid transitional WHO grade 1 meningiomas (DT-G1 meningiomas), we identified an enrichment profile that diverges from the canonical structural and proliferative signatures previously reported by Dai et al. and Cao et al. [20,21]. While inflammatory pathways such as IL-17 and TNF signalling remain detectable, they are overshadowed by prominent metabolic routes, including arachidonic acid metabolism, drug metabolism via cytochrome P450 and retinol metabolism, suggesting a shift toward lipid-mediated signalling and xenobiotic processing in this cytogenetically stable subgroup.

In the WHO CNS5 (2021) classification, traditional histopathological subtypes are preserved, while molecular parameters have been incorporated into the grading framework, refining the current understanding of meningioma biology [22,23]. In accordance with this current scheme, “transitional” remains a valid histopathological subtype and the tumours examined here are interpreted within the WHO grade 1 framework [22,23]. To ensure a biologically coherent framework for the transcriptomic component of this study, the analysis was intentionally restricted to DT-G1 meningiomas, matching the cytogenetic profile of the long-term H8T-MG culture.

The aim of this study was to characterize the molecular programs associated with DT-G1 meningiomas by integrating long-term in vitro models with transcriptomic analyses. We identified DEGs, key metabolic and inflammatory pathways, and central nodes within the protein–protein interaction network to delineate subtype-specific mechanisms. In parallel, we report the establishment and comprehensive immunocytochemical and cytogenetic characterization of grade 1 meningioma cultures maintained under long-term normoxic conditions.

## 2. Materials and Methods

- Establishment of the H8T-MG meningioma cell line derived from a diploid transitional WHO grade 1 tumour (DT-G1); immunocytochemical and cytogenetic characterization

Tumour samples:

The H8T-MG cell line was established from a fresh, viable human meningioma specimen confirmed by routine histopathology as a WHO grade I transitional meningioma according to the 2000 WHO classification [24]. In accordance with contemporary diagnostic standards, the WHO CNS5 (2021) classification has also been cited here as the current official diagnostic framework for CNS tumour taxonomy, and the term “transitional” remains a valid histopathological subtype under this scheme [22,23]. The historical WHO 2000 terminology (“grade_I”) is retained here solely because it reflects the diagnostic criteria in use at the time of surgery.

The tumour originated from the frontal falx of a 41-year-old female patient who underwent surgery at Ribera-Salud Hospital of Alzira (Valencia, Spain). Archival tissue is no longer available, preventing retrospective assessment of WHO 2021 molecular grade-defining biomarkers (TERT-promoter mutation and CDKN2A/B homozygous deletion). The patient remained recurrence-free for more than five years after resection, providing objective clinical evidence of benign grade 1 behaviour.

The H8T-MG and H16T-MG cultures were not newly generated for this study. Both cultures were originally derived between 2003 and 2006 during a funded research project at Universidad Miguel Hernández (UMH), and their derivation and initial characterization are documented in the author’s doctoral thesis [25].

Isolation and culture of primary human meningioma cells:

Fresh tumour tissue was processed immediately after resection in L-15 medium supplemented with B27 (Invitrogen, Carlsbad, CA, USA) and transported on ice. In the laboratory, the sample was dissected into ~1 mm fragments and enzymatically digested with papain and cysteine at 37 °C for 1 h. The suspension was centrifuged at 200× *g* for 5 min, resuspended in DMEM/F12 (Invitrogen, Carlsbad, CA, USA), and mechanically dissociated using a fire-polished Pasteur pipette. After 2 additional centrifugations (200× *g*, 4 min), cells were resuspended in DMEM/F12 containing 10% foetal bovine serum (Sigma-Aldrich, St. Louis, MO, USA) supplemented with 20 ng/mL bFGF, 20 ng/mL EGF and 10 ng/mL LIF (Sigma) and seeded at 1.5 × 10^5^ cells/cm^2^. Cultures were incubated at 37 °C in a humidified 5% CO_2_ atmosphere. Confluent cultures were passaged using 0.25% trypsin/EDTA (Sigma) and medium was replaced weekly.

Growth kinetics:

To determine doubling time, 20,000 cells were seeded in 24-well plates on day 0. Cells were harvested and counted every 24 h for 240 h (24–240 h). At each time point, cells were trypsinized and counted using a Neubauer haemocytometer. Triplicate wells were analysed per time point. Doubling time was calculated from the exponential phase of the growth curve.

Immunocytochemistry:

Immunocytochemistry was performed using standard fixation (4% paraformaldehyde for 20 min at 4 °C), permeabilization, and blocking procedures (PBS with 0.5% Triton X-100 (Sigma-Aldrich, St. Louis, MO, USA), 3% horse serum and 5% BSA for 1 h), with primary and secondary antibodies listed in Supplementary Tables S1 and S2. Nuclei were counterstained with Hoechst 33258 (Sigma-Aldrich, St. Louis, MO, USA; 2 µg/mL) and images were acquired using Zeiss (Oberkochen, Germany) and Leica Microsystems (Wetzlar, Germany) fluorescence microscopes.

Karyotyping:

Exponentially growing cells were treated with 60 µg/mL colchicine (Colcemid; Irvine Scientific, Santa Ana, CA, USA) for 1 h at 37 °C, exposed to hypotonic KCl (0.56%, pH 8.0), and fixed twice with Carnoy’s fixative (3:1 methanol:acetic acid). G-banding was performed using Giemsa staining. Metaphases (n = 10) were analysed using an Olympus BX41 microscope (Olympus, Tokyo, Japan) and CytoVision software (Leica Microsystems, Wetzlar, Germany).

- Establishment of the H16T-MG long-term primary meningioma culture; immunocytochemical characterization

Tumour sample:

The H16T-MG long-term primary culture was established from a fresh transitional meningioma (WHO grade I) resected from the occipital cortex near the torcula of a 34-year-old male patient at Ribera-Salud Hospital of Alzira (Valencia, Spain). Histopathological confirmation followed the 2000 WHO classification criteria. As above, this historical terminology is retained only because the tumour was resected before the introduction of WHO 2016/2021 [22,26]. Archival tissue is no longer available, precluding retrospective assessment of WHO 2021 molecular grade-defining biomarkers. The patient remained recurrence-free for more than five years after surgery.

Isolation and culture conditions:

Cell dissociation and culture procedures were identical to those described for H8T-MG (see above). The H16T-MG culture was maintained for 191 days, showing sustained proliferative capacity without evidence of senescence.

Growth kinetics:

No proliferation curve was performed for H16T-MG, as it represents a long-term primary culture rather than an established cell line.

Immunocytochemistry:

Immunocytochemical was performed using the same antibody panel used for H8T-MG, and H16T-MG cells showed a comparable meningothelial phenotype.

Cytogenetics:

No karyotype analysis was performed for this culture.

- Bioinformatics analysis

All computational analyses were performed in R (version 4.3.3), using packages from Bioconductor (including *limma*, *clusterProfiler*, and *DOSE*) and CRAN (including *EnhancedVolcano*, *pheatmap*, and *igraph*).

Exploratory analysis of raw microarray data

Exploratory evaluation of raw microarray data was performed using hierarchical clustering and principal component analysis (PCA) to assess overall sample structure. Both analyses showed a clear separation between normal meninges and DT-G1 samples.

Differential expression analysis

Publicly available microarray expression data from the Tabernero et al. dataset [10] were used for differential expression analysis. Although the original study includes several histological subtypes and WHO grades of meningioma, the dataset predates WHO 2021 and does not include molecular-grade information. For biological coherence with the H8T-MG culture, only samples of diploid transitional grade 1 without recurrence (39–59 months) were selected for comparison along with normal meninges. Pre-processing and statistical modelling were performed using the *limma* package in R (Ritchie et al., 2015) [27]. Expression values were normalized using quantile normalization, and linear models were fitted for each gene to estimate group differences. Empirical Bayes-moderated statistics were applied, and *p*-values were adjusted for multiple comparisons using the Benjamini–Hochberg false discovery rate (FDR) method. Genes with an adjusted *p*-value (FDR) < 0.05 were considered DEGs.

Visualization of differential expression results

Volcano plots were generated using the *EnhancedVolcano* R package to visualize the distribution and significance of DEGs. Hierarchical clustering and heatmap visualization of DEG expression patterns were performed using the *pheatmap* package.

Functional enrichment analysis

KEGG and GO enrichment analyses were performed using the full set of DEGs obtained from the comparison between normal meninges and DT-G1 samples. Enrichment analyses were conducted using the *clusterProfiler* R package, and results were cross-validated using the DAVID Bioinformatics Resources database [28]. Functional categories and pathways with *p* < 0.05 were considered significantly enriched.

Integration of protein–protein interaction (PPI) network and hub-gene analysis

A protein–protein interaction (PPI) network was uploaded to the STRING database [29], retaining only interactions with a combined confidence score > 0.9. The resulting network was visualized and analysed using the *igraph* package in R [30] and Cytoscape software (version 3.10.2) [31].

Nodes with a connectivity degree > 20 were considered hub genes, representing highly connected nodes that may play central roles in the molecular architecture of DT-G1 meningiomas. Although hub genes were identified, no PPI modules met the MCODE significance criteria (MCODE score > 10 and > 10 nodes); therefore, no module-level enrichment analysis was performed.

## 3. Results

### 3.1. Establishment and Morphological Characterization of the H8T-MG Meningioma Cell Line

#### 3.1.1. Establishment of the Grade 1 Meningioma Cell Line H8T-MG

**Figure 1:**
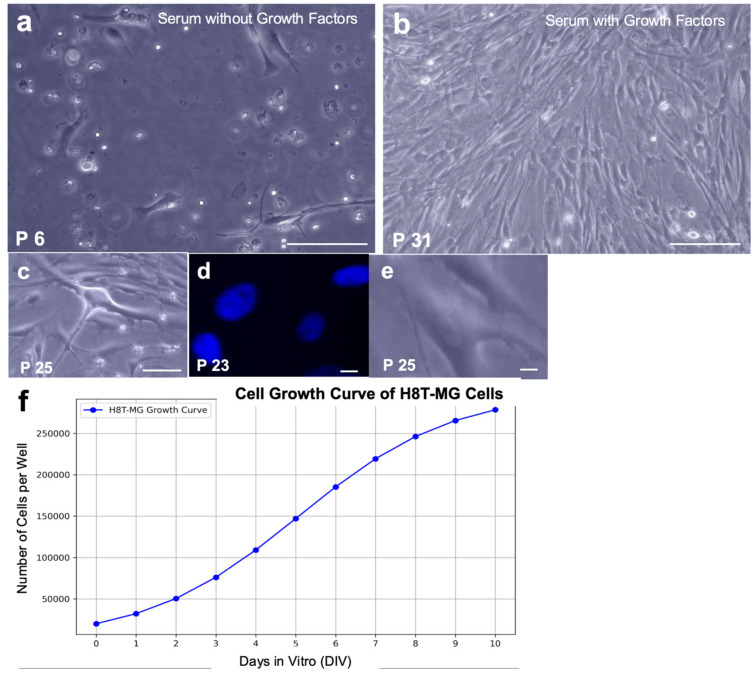
Morphological features and growth behaviour of H8T-MG meningioma cells under different culture conditions. (**a**) Under serum-containing, mitogen-deprived conditions, H8T-MG cells showed a senescent-like phenotype with low density, rounded morphology and loss of adhesion (P6). Scale bar: 200 µm. (**b**,**c**) Under mitogen-supplemented conditions (EGF, bFGF and LIF), cells displayed elongated spindle-shaped morphology and preserved cytoarchitecture across passages (P31, P25). Scale bars: 200 µm (**b**) and 40 µm (**c**). (**d**) DAPI-stained nuclei showing the characteristic elongated/oval morphology under mitogen-supplemented conditions (P23). Scale bar: 10 µm. (**e**) Bright-field image at the same magnification as (**d**), highlighting overall cell morphology and oval nuclei (P25). Scale bar: 10 µm. (**f**) Growth curve of H8T-MG under mitogen-supplemented conditions; estimated doubling time ~40 h.

Primary cultures derived from WHO grade 1 meningiomas showed limited proliferation and enter senescence after a few passages. Under serum-containing conditions without mitogenic stimulation, H8T-MG cells became senescent around passage 8, showing reduced density, enlarged rounded morphology, loss of adhesion and morphological heterogeneity (Figure 1a). In contrast, supplementation with EGF, bFGF and LIF enabled long-term expansion: H8T-MG remained proliferative for over 15 months without detectable signs of senescence (Figure 1b–e). Under these conditions cells adhered rapidly, adopted a spindle-shaped morphology and maintained a stable cytoarchitecture across passages, allowing the establishment of a long-term monolayer culture from WHO grade 1 meningioma without genetic manipulation or hypoxia.

#### 3.1.2. Morphology of H8T-MG Cultures

Under growth factor-supplemented conditions, H8T-MG cells adhered rapidly and proliferated robustly, displaying the elongated, spindle-shaped morphology typical of grade 1 meningioma cultures. This cytological appearance remained stable across passages, with uniform fusiform cells and ovoid nuclei containing prominent nucleoli (Figure 1b–e).

#### 3.1.3. Growth Kinetics

H8T-MG cells showed stable proliferative behaviour under mitogen-supplemented conditions, characterized by adherent growth in monolayer cultures. The growth curve followed a sigmoidal pattern with an initial lag phase, a clear exponential phase, and a plateau reflecting growth arrest upon confluence (Figure 1f), consistent with the preservation of contact inhibition.

The estimated population doubling time was ~40 h, based on the 10-day growth assay shown in Figure 1f, indicating controlled proliferative kinetics under assay conditions.

#### 3.1.4. Immunocytochemical Characterization of H8T-MG

**Figure 2:**
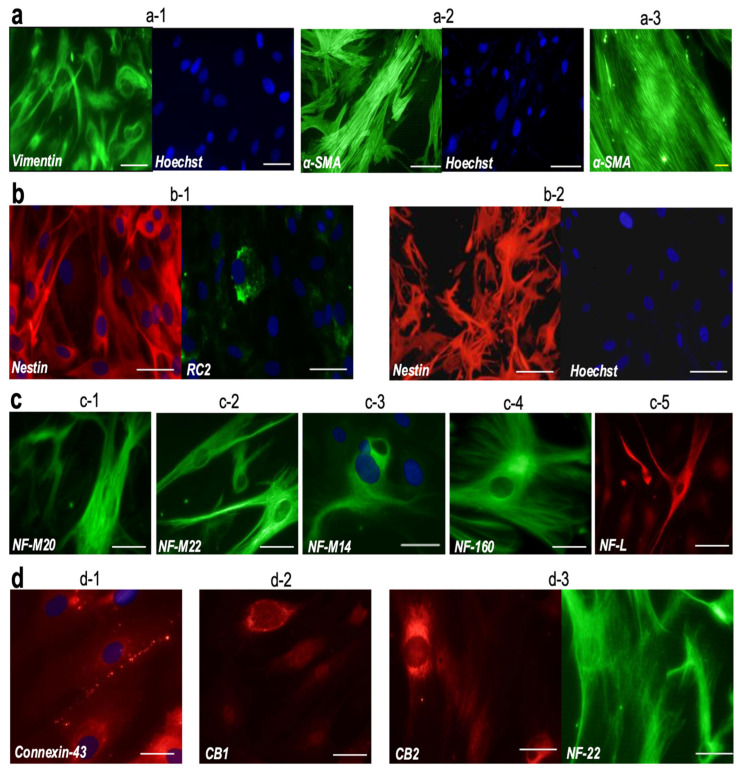
Immunocytochemical characterization of grade 1 meningioma cultures H8T-MG and H16T-MG. (**a**–**d**) Representative immunostainings for vimentin, α-SMA, nestin, RC2, neurofilament subunits (NF-M20, NF-M22, NF-M14, NF-160, NF-L), connexin-43, CB1 and CB2. H8T-MG images correspond to panels (**a-1**,**a-2**,**b-1**,**c-2**,**c-4**,**c-5**,**d-3**); H16T-MG images correspond to panels (**a-3**,**b-2**,**c-1**,**c-3**,**d-1**,**d-2**). All markers were detected in both cultures; only representative images are shown. White scale bars: 50 µm. Yellow scale bars: 15 µm.

To characterize the immunophenotypic profile of the long-term H8T-MG cultures, we performed an immunocytochemical panel covering mesenchymal, epithelial/neural-associated and additional lineage markers. As expected for meningioma-derived cells, H8T-MG showed strong mesenchymal profile: vimentin was expressed in most cells (95.07 ± 2.86%) (Figure 2(a-1)), and α-smooth muscle actin (α-SMA) positivity was also high (77.34 ± 1.57%) (Figure 2(a-2)).

**Figure 3:**
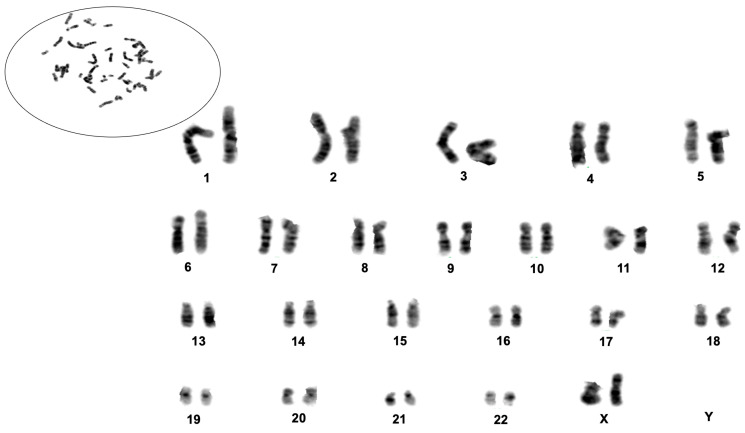
G-banded karyotype analysis of the H8T-MG meningioma culture. Representative metaphase spread showing a normal distribution of chromosomes without visible fragmentation or gross structural abnormalities. Ordered karyotype obtained from G-banded metaphases, confirming the absence of major numerical or structural chromosomal alterations in H8T-MG cells.

**Figure 4:**
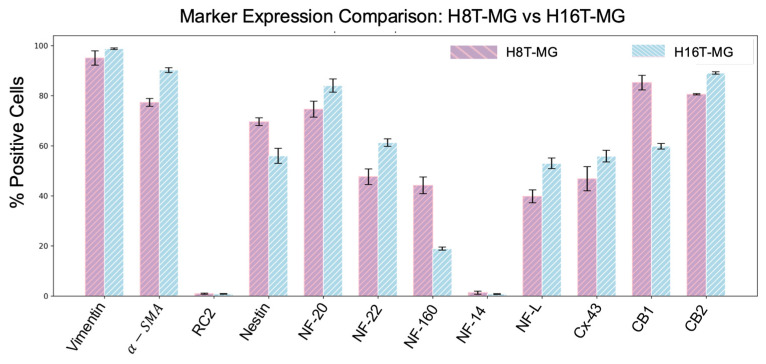
Quantitative comparison of immunocytochemical marker expression in H8T-MG and H16T-MG cultures. Bars show the mean percentage of immunopositive cells ± SD. Lilac/pink bars represent H8T-MG and light-blue/white bars represent H16T-MG. Markers include vimentin, α-SMA, nestin, RC2, neurofilaments, Cx-43, CB1 and CB2.

**Figure 5:**
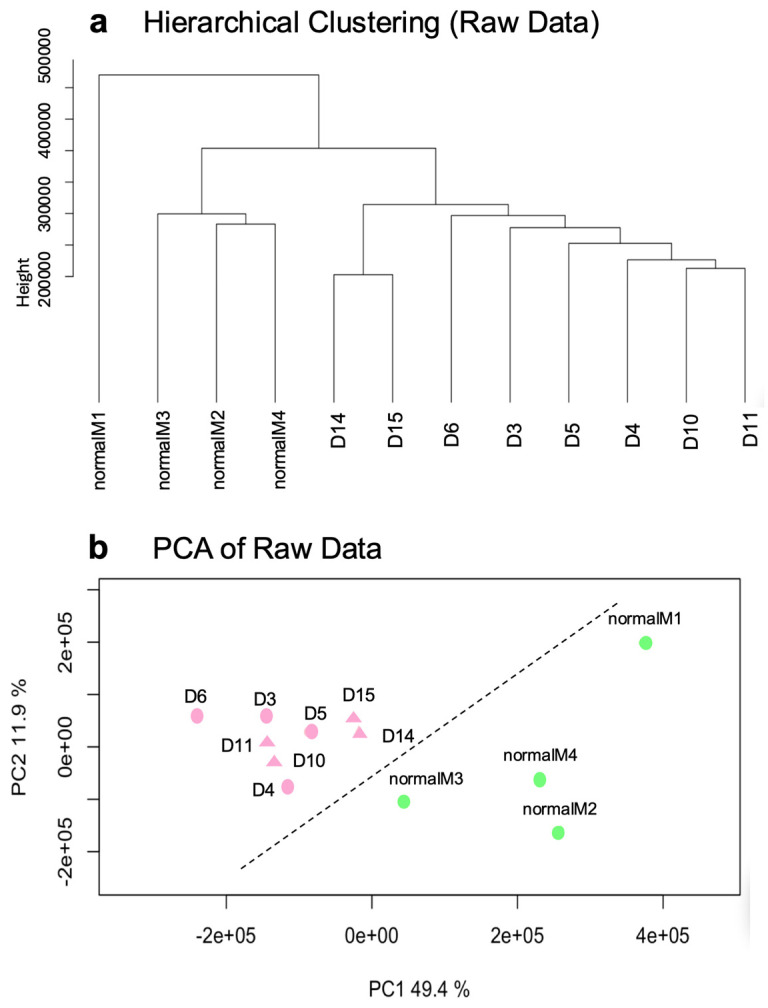
Exploratory analysis of raw microarray data. (**a**) Hierarchical clustering of unprocessed expression values showing two distinct sample groups corresponding to normal meninges and DT-G1 meningiomas. (**b**) Principal component analysis (PCA) of raw data. PC1 and PC2 together explain 61.3% of the variance and position meningioma samples and control meninges in clearly separate regions of the plot (figure panels generated using the dataset described in Supplementary Table S3).

Among epithelial and neural-associated markers, nestin was detected in a substantial fraction of cells (69.61 ± 1.57%), whereas the radial glia-associated marker RC2 appeared only in a small subset (0.91 ± 0.21%) (Figure 2(b-1)). H8T-MG also showed robust immunoreactivity for multiple neurofilament subunits, including medium-weight (NF-M20, NF-M22, NF-M14) and low-weight (NF-L) isoforms, indicating a broad neural-associated intermediate filament profile (Figure 2(c-2,c-4,c-5)).

Additionally, cells displayed strong positivity for connexin-43 (46.83 ± 4.83%), consistent with gap-junction expression in meningothelial-derived cultures. An abundant expression of cannabinoid receptors CB1 and CB2 (85.23 ± 2.87% and 80.57 ± 0.23%) was observed, highlighting the presence of signalling receptors whose expression has been only sparsely documented in grade 1 meningioma cultures (Figure 2(d-3)).

#### 3.1.5. Karyotyping of H8T-MG

Conventional G-banded karyotyping was performed on exponentially growing H8T-MG cells to evaluate chromosomal stability. Analysis of 10 metaphases revealed no large-scale abnormalities. Metaphase spreads showed a normal chromosome distribution without fragmentation, dicentric figures or overt aneuploidies (Figure 3). The ordered karyotype confirmed the absence of major numerical or structural alterations, indicating that H8T-MG maintains a stable chromosomal complement under prolonged mitogen-supplemented culture conditions.

### 3.2. Characterization of the H16T-MG Long-Term Primary Culture

A second transitional WHO grade 1 meningioma culture, H16T-MG, was established under the same growth factor-supplemented conditions used for H8T-MG. Under these conditions, H16T-MG remained viable and proliferative for 191 days before entering senescence. Throughout the culture period, cells displayed elongated, fibroblast-like morphology with fusiform cells and oval nuclei (comparable to H8T-MG), exhibited stable adherent growth in monolayer cultures, and ceased proliferation upon reaching confluence. No proliferation curve or karyotype analysis was performed for this culture.

Immunocytochemical profiling revealed a meningothelial phenotype comparable to that of H8T-MG. H16T-MG showed high expression of mesenchymal markers, including vimentin (98.80 ± 0.27%) and α-SMA (90.24 ± 0.97%) (Figure 2(a-3)). Neural-associated markers were also detected, although at different levels from H8T-MG: nestin was present in 55.97% ± 2.99 whereas RC2 remained restricted to a very small subset (0.84 ± 0.14%) (Figure 2(b-2)).

Consistent with H8T-MG, H16T-MG cells displayed a broad expression of neurofilament subunits, including NF-M20 (84.04% ± 2.64), NF-M22 (61.28% ± 1.50), NF-160 (18.91 ± 0.61%) and NF-M14 (0.76 ± 0.16%) (Figure 2(c-1,c-3)). Additional markers such as connexin-43 (55.85 ± 2.33%), CB1 (59.82 ± 1.12%) and CB2 (89.07 ± 0.46%) were strongly expressed, closely mirroring the profile observed in H8T-MG (Figure 2(d-1,d-2)).

Importantly, a direct quantitative comparison between both cultures (Figure 4) showed that H8T-MG and H16T-MG share a highly consistent immunocytochemical signature, despite differences in their long-term proliferative lifespan.

### 3.3. Bioinformatics Analysis of Transcriptomic Differences Between Normal Meninges and DT-G1 Meningiomas

#### 3.3.1. Exploratory Analysis of Raw Microarray Data

Exploratory analysis was performed to assess sample relationships prior to normalization. The dataset contained four normal meningeal samples and three DT-G1 meningiomas (Supplementary Table S3). Hierarchical clustering of raw expression values separated the samples into two distinct groups, with all meninges clustering together and apart from the meningiomas (Figure 5a).

Principal component analysis (PCA) confirmed this pattern: the first two principal components explained 61.3% of the total variance and clearly positioned normal meninges and tumour samples in different regions of the plot (Figure 5b). These results indicate that, even before normalization, the raw dataset exhibit well-defined transcriptional differences between healthy meninges and DT-G1 meningiomas.

#### 3.3.2. Normalization and Quality Assessment of Processed Microarray Data

Following exploratory analysis, raw intensities were normalized to reduce technical variability and improve comparability across samples. Normalization decreased dispersion while preserving biological differences between groups. Post-normalization sample distances and clustering continued to segregate DT-G1 meningioma samples from healthy meninges confirming the robustness of preprocessing workflow and the suitability of the dataset for downstream differential expression analysis.

#### 3.3.3. Differential Expression Overview

We compared four healthy meningeal samples with eight DT-G1 meningioma samples (Supplementary Table S3) using the limma workflow in R. After normalization and filtering, 8130 genes were retained. Differential expression analysis identified 51 DEGs, corresponding to 61 ProbeIDs, some of which were represented by multiple ProbeIDs. Most DEGs were downregulated in meningiomas relative to healthy meninges, whereas only 10 genes were significantly upregulated (Table 1).

#### 3.3.4. Differential Expression Patterns Visualized by Volcano Plot

**Figure 6:**
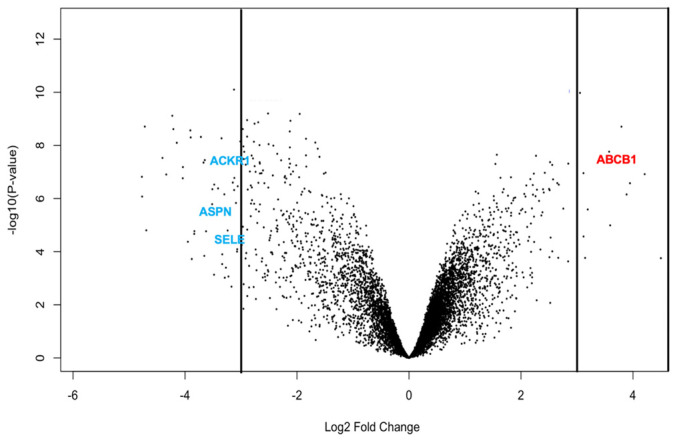
Volcano plot of differentially expressed genes between DT-G1 meningiomas and healthy meninges. The plot shows log_2_ fold changes versus −log_10_ adjusted *p*-values for all transcripts. Significantly upregulated genes appear on the right, and downregulated genes on the left, with non-significant genes centred around log_2_FC = 0. Four key hub genes—*ASPN*, *SELE*, *ABCB1*, and *ACKR1*—are highlighted due to their strong deregulation and relevance in grade 1 meningioma biology.

The volcano plot (Figure 6) shows the global distribution of log_2_ fold changes versus significance. A clear asymmetry was observed, with many more significantly downregulated transcripts and a smaller cluster of upregulated genes. Among the deregulated genes ASPN, SELE, ACKR1, and ABCB1 stood out by combining strong changes (|log_2_FC| > 3) with high connectivity in the PPI network, highlighting them as biologically relevant hub genes (see hub analysis below).

#### 3.3.5. Hierarchical Clustering of Differentially Expressed Genes

**Figure 7:**
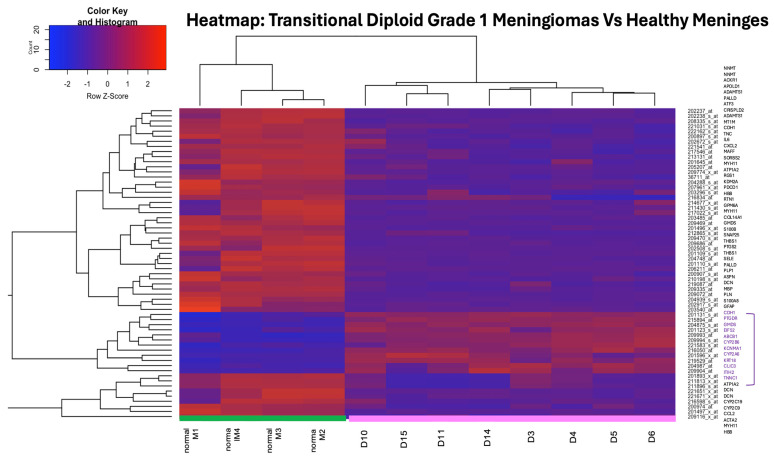
Hierarchical clustering heatmap of DEGs. Heatmap showing hierarchical clustering of DEGs between DT-G1 meningiomas and healthy meninges. Normalized expression values were clustered using average linkage, and the colour gradient represents relative expression levels. Healthy samples (green) and meningiomas (pink) form two clearly separated clusters, highlighting distinct tumour-associated transcriptional profiles.

Hierarchical clustering of the significantly deregulated genes confirmed the clear transcriptional separation between groups (Figure 7). Healthy meninges and DT-G1 meningiomas formed two robust and distinct clusters, reflecting highly consistent within-group expression patterns. Genes grouped into coherent blocks of up- and downregulation, reinforcing the biological relevance of the DEGs and indicating a stable disease-associated transcriptional signature.

#### 3.3.6. Gene Ontology Enrichment Analysis

**Figure 8:**
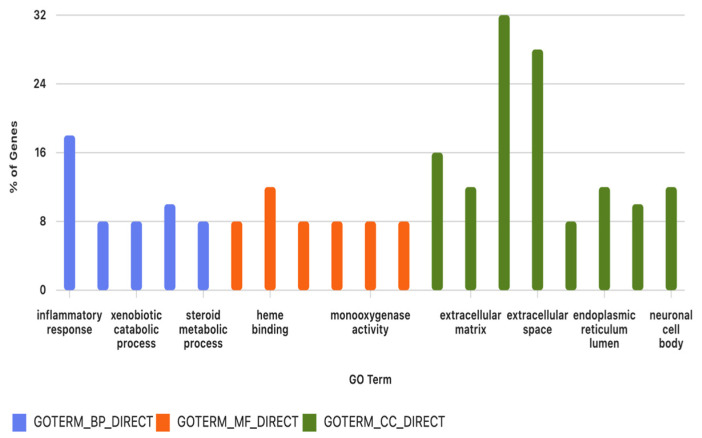
GO enrichment analysis of DEGs. Bar plot showing the most significantly enriched GO among the 51 DEGs. Bar height reflects the percentage of associated genes, and bar colour indicates GO category (BP, MF, or CC). Enriched terms relate mainly to inflammatory response, extracellular matrix organization, xenobiotic metabolism, oxidoreductase activity, and extracellular region components, highlighting key pathways altered in DT-G1 meningiomas. (GO: Gene Ontology; BP: Biological process; MF: Molecular function; CC: Cellular component).

GO over-representation analysis revealed enrichment of terms related to inflammatory response, collagen-containing extracellular matrix, extracellular region/space, xenobiotic metabolic and catabolic processes, heme binding/oxidoreductase activity, and actin cytoskeleton-related structures, among others (Supplementary Table S4; Figure 8). These categories point to alterations in immune-inflammatory pathways, extracellular matrix remodelling, and xenobiotic and lipid-associated enzymatic functions in DT-G1 meningiomas.

#### 3.3.7. KEGG Pathway Enrichment Analysis

**Figure 9:**
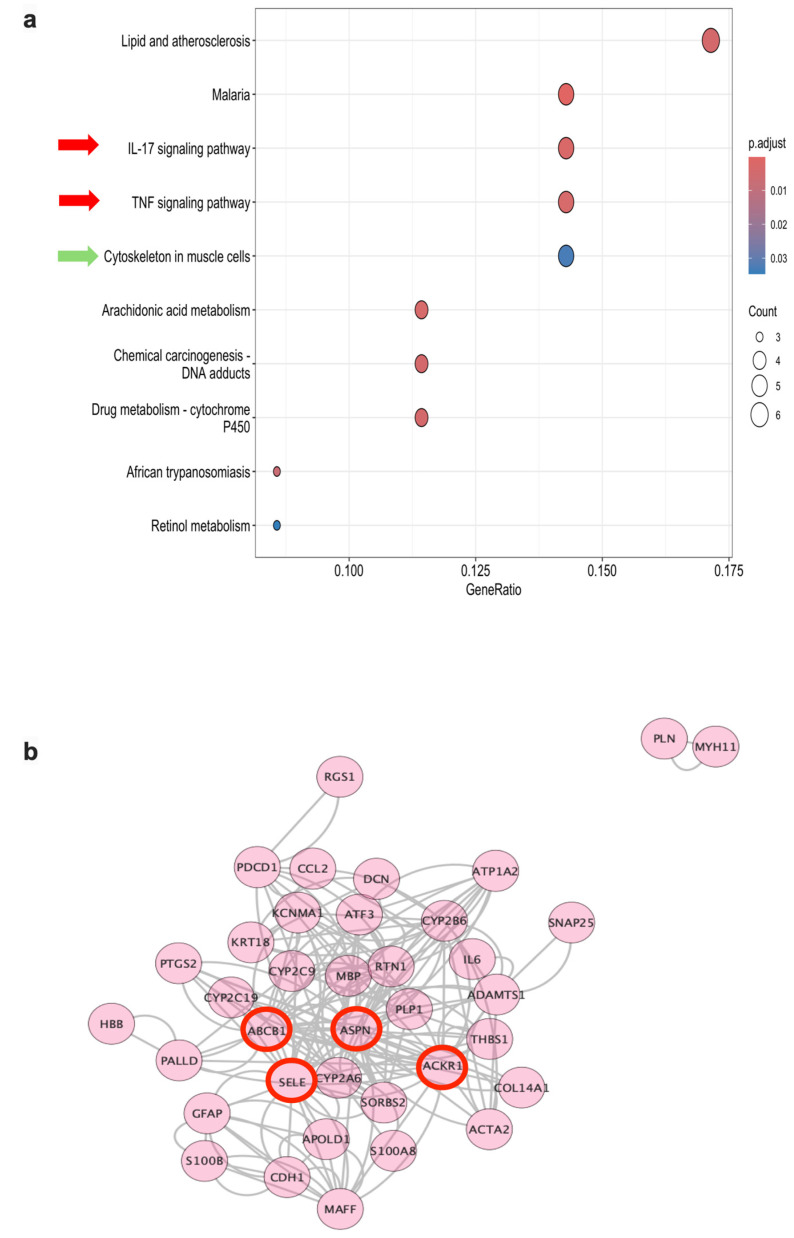
KEGG pathway enrichment and PPI network of DEGs in DT-G1 meningiomas. (**a**) Dot plot of significantly enriched KEGG pathways ranked by adjusted *p*-value; dot colour indicates significance and dot size represents the gene ratio. (**b**) PPI network generated from the 51 DEGs using STRING, showing 37 nodes and 180 edges. Hub genes with the highest connectivity (*ASPN*, *SELE*, *ABCB1*, *ACKR1*) are highlighted. No significant MCODE clusters were detected.

KEGG over-representation analysis identified 10 significantly enriched pathways (Table 2; Figure 9a), including the following:

- IL-17 signalling pathway;
- TNF signalling pathway;
- Cytoskeleton in muscle cells;
- Arachidonic acid metabolism;
- Lipid and atherosclerosis;
- Drug metabolism—cytochrome P450;
- Chemical carcinogenesis—DNA adducts;
- Retinol metabolism;
- Malaria (shared inflammatory–vascular component);
- African trypanosomiasis (shared inflammatory–vascular component).

**Table 2:** KEGG pathway enrichment analysis of differentially expressed genes in DT-G1 meningiomas compared with healthy meninges.

Category	Subcategory	Description	*p*-Value	*p*-Adjust	Count	GeneID *(NCBI)*	Gene Symbol	Direction of Gene Deregulation Benign Transitional Diploid Meningioma vs. Meninges
Human Diseases (hsa05144)	Infectious disease: parasitic	*Malaria*	1.347 × 10^−6^	0.0001953688	5	6347/3043/6401/7057/3569	CCL2	↓ in Meningiomas Grade 1
							HBB	↓ in Meningiomas Grade 1
							SELE	↓ in Meningiomas Grade 1
							THBS1	↓ in Meningiomas Grade 1
							IL6	↓ in Meningiomas Grade 1
Organismal Systems (hsa04657)	Immune system	*IL-17 signaling pathway*	3.243 × 10^1^	0.0023512379	4	6347/5743/3569/6279	CCL2	↓ in Meningiomas Grade 1
							PTGS2	↓ in Meningiomas Grade 1
							IL6	↓ in Meningiomas Grade 1
							S100A8	↓ in Meningiomas Grade 1
Metabolism (hsa00590)	Lipid metabolism	*Arachidonic acid metabolism*	9.156 × 10^1^	0.0034617933	4	1555/1557/1558/5743	CYP2B6	↑ in Meningiomas Grade 1
							CYP2C19	↓ in Meningiomas Grade 1
							CYP2C9	↓ in Meningiomas Grade 1
							PTGS2	↓ in Meningiomas Grade 1
Environmental Information Processing (hsa04668)	Signal transduction	*TNF signaling pathway*	9.550 × 10^1^	0.0034617933	4	6347/5743/6401/3569	CCL2	↓ in Meningiomas Grade 1
							PTGS2	↓ in Meningiomas Grade 1
							SELE	↓ in Meningiomas Grade 1
							IL6	↓ in Meningiomas Grade 1
Human Diseases (hsa05204)	Cancer: overview	*Chemical carcinogenesis - DNA adducts*	1.658 × 10^2^	0.0038248597	4	1548/1557/1558/5743	CYP2A6	↑ in Meningiomas Grade 1
							CYP2C19	↓ in Meningiomas Grade 1
							CYP2C9	↓ in Meningiomas Grade 1
							PTGS2	↓ in Meningiomas Grade 1
Human Diseases (hsa05417)	Cardiovascular disease	*Lipid and atherosclerosis*	1.781 × 10^2^	0.0038248597	6	1555/1548/1558/6347/6401/3569	CYP2B6	↑ in Meningiomas Grade 1
							CYP2A6	↑ in Meningiomas Grade 1
							CYP2C9	↓ in Meningiomas Grade 1
							CCL2	↓ in Meningiomas Grade 1
							SELE	↓ in Meningiomas Grade 1
							IL6	↓ in Meningiomas Grade 1
Metabolism (hsa00982)	Xenobiotics biodegradation and metabolism	*Drug metabolism-cytochrome P450*	1.846 × 10^2^	0.0038248597	4	1555/1548/1557/1558	CYP2B6	↑ in Meningiomas Grade 1
							CYP2A6	↑ in Meningiomas Grade 1
							CYP2C19	↓ in Meningiomas Grade 1
							CYP2C9	↓ in Meningiomas Grade 1
Human Diseases (hsa05143)	Infectious disease: parasitic	*African trypanosomiasis*	4.029 × 10^2^	0.0073022271	3	3043/6401/3569	HBB	↓ in Meningiomas Grade 1
							SELE	↓ in Meningiomas Grade 1
							IL6	↓ in Meningiomas Grade 1
*NA* (hsa04820)	*NA*	*Cytoskeleton in muscle cells*	2.035 × 10^3^	0.0327837258	5	7134/7057/478/4629/1634	TNNC1	↑ in Meningiomas Grade 1
							THBS1	↓ in Meningiomas Grade 1
							ATP1A2	↓ in Meningiomas Grade 1
							MYH11	↓ in Meningiomas Grade 1
							DCN	↓ in Meningiomas Grade 1
Metabolism (hsa00830)	Metabolism of cofactors and vitamins	*Retinol metabolism*	2.389 × 10^3^	0.0346464396	3	1555/1548/1558	CYP2B6	↑ in Meningiomas Grade 1
							CYP2A6	↑ in Meningiomas Grade 1
							CYP2C9	↓ in Meningiomas Grade 1

Notes: KEGG categories and pathway names follow the KEGG database. *p*-value indicates unadjusted significance; p-adjust corresponds to Benjamini–Hochberg correction. Count is the number of DEGs mapped to each pathway, and GeneID refers to NCBI identifiers. Upward arrows (↑) indicate genes upregulated in DT-G1 meningiomas, while downward arrows (↓) indicate downregulated genes relative to healthy meninges.

These pathways collectively reflect alterations in inflammatory signalling, cytoskeletal organization, and xenobiotic/lipid metabolism.

Figure 9 shows the KEGG enrichment dot plot and the corresponding PPI network derived from the significantly deregulated genes.

Among the enriched pathways, IL-17 and TNF signalling showed the lowest adjusted *p*-values, and both were downregulated in meningiomas compared with healthy meninges, consistent with reduced pro-inflammatory and immune-reactive activity. The ‘Cytoskeleton in muscle cells’ pathway contained five DEGs, four downregulated (THBS1, MYH11, ATP1A2, DCN) and one upregulated (TNNC1), indicating altered cytoskeletal and contractile processes in tumour cells (Table 2; Figure 9a).

Together, these findings suggest that DT-G1 meningiomas adopt a less inflammatory, and more immune-tolerant microenvironment, accompanied by structural remodelling and altered lipid and xenobiotic metabolism.

#### 3.3.8. PPI Network and Hub Gene Identification

A STRING-derived PPI network built from the 51 DEGs included 37 nodes and 180 edges (Figure 9b). Network topology analysis identified four hub genes (Table 3):

**Table 3:** Hub genes identified in the PPI network, their degree, regulation status and main function.

Gene Symbol	Description	Degree	Regulation	Main Function
ASPN	*Asporin*	30	↓	Extracellular matrix organisation; collagen binding
SELE	*E-selectin*	26	↓	Endothelial adhesion; inflammatory cell recruitment
ABCB1 (=MDR1)	*ATP-binding cassette sub-family B member 1* *(P-glycoprotein 1)*	24	↑	ATP-dependent efflux transporter; xenobiotic/drug export
ACKR1 (=DARC)	*Atypical chemokine receptor 1 (Duffy antigen receptor for chemokines*	22	↓	Chemokine scavenging; immune-vascular modulation

Notes: Hub genes identified in the PPI network generated from DEGs. Degree indicates the number of direct interactions per node in the STRING network. Upward arrows (↑) denote genes upregulated in DT-G1 meningiomas, and downward arrows (↓) denote downregulated genes.

These genes represent key biological processes in grade 1 meningioma.

ASPN → Extracellular matrix organization and collagen binding.

SELE → Endothelial adhesion and inflammatory cell recruitment.

ACKR1 (DARC) → Chemokine scavenging and immune–vascular modulation.

ABCB1 (MDR1) → ATP-dependent efflux and xenobiotic/drug export.

Expression analysis (Table 1) confirmed that ABCB1 was upregulated, whereas ASPN, SELE, and ACKR1 were downregulated (Figure 6). Although other DEGs showed higher statistical significance, these four genes combined strong deregulation (|log_2_FC| > 3) with high PPI connectivity. Together, these features identify them as key hub genes in DT-G1 meningioma biology.

MCODE analysis did not identify dense submodules (score > 10 or >10 nodes), indicating a network composed of interconnected but non-clustered functional nodes, rather than tightly defined molecular complexes.

## 4. Discussion

WHO grade 1 meningiomas are the most common primary intracranial tumours, yet a substantial subset recurs after surgery, with reported recurrence rates of 7–23% at 5 years [32,33]. These observations underscore the need for biologically faithful in vitro models and subtype-specific genomic analyses to better understand early disease biology.

In this study, we established and characterized a grade 1 meningioma cell line (H8T-MG) and complemented it with an independently derived long-term primary culture (H16T-MG). H8T-MG provided the experimental foundation for our cytogenetic and molecular characterization, whereas H16T-MG was used as independent support for in vitro morphology, growth behaviour and to demonstrate that transitional grade 1 cells can be maintained long-term in vitro.

We further integrated these experimental data with a subtype-restricted transcriptomic dataset consisting exclusively of DT-G1 meningiomas, matching the cytogenetic and histological profile of the H8T-MG cell line. Together, these analyses support the identification of a reproducible inflammatory–metabolic program associated with DT-G1 meningiomas and provide a biologically coherent, hypothesis-generating framework for interpreting early molecular processes involved in the development of this subtype.

According to the recent WHO CNS5 (2021), TERT-promoter mutation and homozygous CDKN2A/B deletion define WHO grade 3 irrespective of tumour histology [22,23]. Interpreting our models within this framework, multiple clinical and cytogenetic features indicate that these alterations are unlikely to be present in our tumours.

First, H8T-MG displays a strictly diploid karyotype, and the transitional grade 1 cases selected from the Tabernero dataset are also diploid [10]. CDKN2A/B homozygous deletion is strongly associated with chromosomal instability and is virtually absent in diploid grade 1 tumours [34,35,36]. Second, TERT-promoter mutations are rare (<5% across grades 1–3) and occur predominantly in tumours with extensive copy-number complexity. Multi-institutional cohorts show that TERTp-mutant tumours exhibit high chromosomal instability and frequent 1p and CDKN2A/B loss [37,38,39], patterns incompatible with the strictly diploid background of H8T-MG and of the matched transcriptomic subset. Third, both donor tumours remained recurrence-free for >5 years, a clinical trajectory inconsistent with TERT-mutant or CDKN2A/B-deleted meningiomas, which typically recur early [10,33,34,37,40]. This long-term stability aligns instead with cytogenetically stable grade 1 disease.

Taken together, these observations support the interpretation of both H8T-MG and the diploid transitional tumours from the Tabernero dataset within a WHO 2021 grade 1 biological framework [10].

Only one grade 1 meningioma culture has previously been established without genetic manipulation, TKB-MEN2 [16], but its maintenance required hypoxia (3% O_2_). By comparison, our system sustained long-term proliferation under normoxic conditions supplemented with EGF, bFGF and LIF, which is biologically plausible given the role of these pathways in neural progenitor division [41,42] and the high prevalence of EGFR expression in meningiomas (~89%) [43].

H8T-MG maintained long-term, controlled proliferative behaviour (>1 year) with a population doubling time of ~40 h, characterized by adherent growth in monolayer cultures and growth arrest upon reaching confluence, features that are consistent with WHO grade 1 meningioma cell models [13,14,16] and distinct from high-grade meningioma lines, which typically display accelerated proliferation, multilayered growth and frequent chromosomal instability [44,45,46].

H16T-MG also proliferated (191 days) before entering senescence, exhibiting comparable morphological and growth characteristics, supporting the robustness of the culture system, although its non-karyotyped status limits genomic interpretation.

Based on these features, H8T-MG can be considered an established DT-G1 meningioma cell line, whereas H16T-MG corresponds to a long-term primary culture. The phenotypic similarities between both models reflect their shared immunocytochemical profile rather than genomic equivalence.

Importantly, the use of an independently derived culture (H16T-MG) displaying highly concordant morphological, growth and immunocytochemical characteristics provides internal biological support for the relevance of the H8T-MG model.

Together, these two cultures provide a normoxic, genetically unmanipulated in vitro framework for studying transitional grade 1 meningiomas. Long-term, biologically stable cultures are essential for studying tumour differentiation and for generating in vitro systems that reflect disease biology. Most available models rely either on malignant meningioma lines or on grade 1 lines that have been genetically immortalized such as Ben-Men-1 [13]. Only one non-manipulated grade 1 culture, TKB-MEN2, has been maintained long-term, although it requires hypoxia [16]. By comparison, our models allow the expansion of grade 1 meningioma cells under normoxia, preserving their characteristic immunocytochemical features, and thereby providing a biologically coherent platform for molecular and functional studies.

This reinforces the relevance of analysing their immunocytochemical profiles, as these cultures represent biologically faithful transitional grade 1 models. An essential step in tumour studies is the in vitro characterization of cultured cells, and this becomes more informative when complemented with transcriptomic profiles. Generally, there is a correlation between marker expression in tumour tissue and in the corresponding cultures [47]. Importantly, meningiomas, unlike other CNS tumour types, derive from a mixed meso-neuroectodermal lineage, exhibiting a characteristic dual mesenchymal and epithelial differentiation potential [48]. In our cultures, this identity was clearly evident, with several markers matching previous reports and others not previously described, supporting both the fidelity and novelty of our models.

### 4.1. Immunocytochemical Characterization of H8T-MG and H16T-MG

Both H8T-MG and H16T-MG displayed a convergent meningothelial phenotype, characterized by robust expression of mesenchymal markers (vimentin, α-SMA) and epithelial/neural-associated markers (RC2, nestin, and multiple neurofilament subunits), reflecting the mixed meso-neuroectodermal origin of meningothelial cells [48,49]. This dual epithelial–mesenchymal identity, a hallmark of arachnoid-derived tissues, supports the biological fidelity of our long-term cultures.

Importantly, preservation of meningothelial identity in vitro is not defined by isolated lineage marker expression, but by the coordinated presence of mesenchymal and ecto/neuroectodermal features together with organized intercellular junctions.

In this context, the concurrent expression of vimentin, α-SMA, neural-associated intermediate filaments and junction-related proteins observed in our cultures reflects a coherent meningothelial pattern rather than heterogeneous or dedifferentiated marker expression.

Classical benign WHO grade 1 meningioma models have been validated on the basis of concurrent vimentin expression and junctional proteins, such as desmoplakin, reflecting preserved cell–cell adhesion without requiring uniform marker expression across the entire cell population [14].

Accordingly, the immunocytochemical profile observed in H8T-MG and H16T-MG follow established biological criteria accepted in the field for meningioma culture validation.

Vimentin, a hallmark of meningothelial differentiation, was expressed in >95% of H8T-MG cells, in line with previous grade 1 meningioma cultures [13,14,44,46,50,51]. H16T-MG showed a similarly high expression level, reinforcing the notion that the strong mesenchymal backbone of H8T-MG is a consistent feature of DT-G1 meningiomas.

α-SMA, associated with normal and tumour-associated myoepithelial/meningothelial lineages [52,53,54,55], was robustly expressed in both models. Immunocytochemistry showed α-SMA positivity in 77.34% ± 1.57 in H8T-MG cells, consistent with transitional meningioma cytoskeletal features. H16T-MG displayed even higher α-SMA expression (90.24% ± 0.97) consistent with preservation of myoepithelial/meningothelial differentiation features in vitro.

Nestin, rarely reported in monolayer grade 1 cultures and typically linked to neurosphere-derived stem-like fractions [56], was prominently expressed in both cultures (~70% of H8T-MG, comparable levels in H16T-MG). This suggests that transitional meningioma cells may retain or reacquire progenitor-like or stress-responsive features in vitro. RC2, a classical radial glia marker [57], appeared only in a very small minority of cells, consistent with its developmental restriction.

Medium- and light-chain neurofilaments, established neuronal cytoskeletal proteins, also showed clear immunoreactivity in both cultures. Although variably documented in grade 1 meningiomas, their presence here indicates preservation of neuroectodermal features, reinforcing the epithelial/neural component of the meningothelial identity.

Connexin-43, a gap-junction protein abundant in glial, arachnoid and vascular tissue [58,59,60], also showed strong immunopositivity (~47% in H8T-MG; similar in H16T-MG), consistent with the junction-rich ultrastructure of meningothelial cells.

Cannabinoid receptors CB1 and CB2, previously reported in gliomas and in vivo meningiomas but never before in grade 1 cultures, were robustly expressed in both models (CB1 ~85%, CB2 ~80%) [61,62,63]. Given the reported antitumour and anti-angiogenic roles of cannabinoid signalling [64,65,66,67,68], their presence represents a novel finding that warrants further investigation.

Minor quantitative differences between H8T-MG and H16T-MG likely reflect donor-specific variation. Both cultures underwent the same antibody panel and quantification, yielding highly comparable mesenchymal, epithelial/neural, cannabinoid receptors and junction-associated expression profiles. This supports the biological robustness of the culture system rather than idiosyncratic variation.

The immunocytochemical profile of both models provides a coherent framework for interpreting the transcriptomic programs identified in DT-G1 meningiomas. Preservation of mesenchymal and neuroectodermal features supports the biological relevance of the metabolic and inflammatory pathways highlighted in tumour tissue.

Consistently, although α-SMA is detectable in vitro, ACTA2 is downregulated in tumour tissues, suggesting that the in vivo microenvironment of DT-G1 meningiomas is low-tension and characterized by reduced cytoskeletal activity, features that align with a low-remodelling ECM and immune-cold landscape.

Taken together, these phenotypic and functional criteria are consistent with established approaches historically used for meningioma culture validation [13,14,18].

Building on this phenotypic characterization, we next examined whether molecular programs in tumour tissue align with these in vitro findings. By identifying DEGs between tumours and normal meninges, we aim to elucidate the microenvironmental influences shaping early DT-G1 meningioma biology.

Recent expert reviews emphasize that transcriptomic signals emerge most clearly when analyses are restricted to biologically homogeneous subgroups defined by histology, cytogenetic stability and clinical behaviour [11,69,70,71]. By focusing exclusively on non-recurrent DT-G1 meningiomas, both in vitro (H8T-MG) and in the selected cases from the Tabernero dataset [10], we minimized the confounding effects of biological heterogeneity. This stratified approach facilitates the identification of subtype-specific molecular programs to emerge that would otherwise remain obscured in pooled datasets.

### 4.2. Transcriptomic Analysis

To date, only two studies have compared meningioma tissue with normal meninges using microarray-based transcriptomic approaches: The work of Dai et al. (2017), which included WHO grades 1–3 [20], and that of Cao et al. (2020), which focused on grade 1 tumours across all histological subtypes [21]. Our analysis adds a refined perspective by restricting the comparison to DT-G1 meningiomas, thereby offering a more focused subtype-specific view of early-stage biology.

Consistent with these two studies, most DEGs in our cohort were downregulated, suggesting that early grade 1 meningiomas are characterized by limited broad pathway activation together with a predominance of selective gene silencing, particularly when interpreting the GO and KEGG enrichments results. Table 4 compiles the principal functional and structural pathways described in prior grade 1 studies [20,21] and contrasts them with our transitional subtype signature.

#### 4.2.1. GO Enrichment Analysis

Our GO enrichment analysis identified both overlaps and differences with the transcriptomic profiles reported by Dai et al. (2017) and Cao et al. (2020) (Table 4) [20,21]. Across the three comparisons, we observed downregulation of processes related to ‘extracellular matrix organization’ and ‘cell adhesion’, although these terms reached only modest significance in our cohort, likely due to the narrower focus on DT-G1 meningiomas.

We found a marked downregulation of genes related to inflammatory response (IL6, CXCL2, CCL2) and extracellular matrix organization (DCN, COL14A1, THBS1, ASPN, ADAMTS1). This pattern is consistent with attenuated immune signalling and reduced structural remodelling. It aligns with Cao et al. (2020) [21] and with evidence that grade 1 meningiomas show attenuated immune activation and altered matrix dynamics compared with higher-grade tumours, features that may influence microenvironmental modulation and recurrence risk [72,73]. Similarly, the reduced expression of calcium-binding proteins such as S100B and S100A8 (Table 1) points to diminished calcium-dependent signalling pathways, which affect cell adhesion, cytoskeletal organization and motility. Alterations in these processes have been associated with tumour behaviour and progression, adding another layer of microenvironmental modulation consistent with transcriptomic observations in meningioma [74].

Notably, several GO categories were uniquely enriched in our comparison, including ‘xenobiotic metabolism’, ‘epoxygenase activity’, ‘arachidonate epoxygenase activity’, ‘oxidoreductase activity’ and ‘monooxygenase activity’ (Table 4). These pathways are indicative of a metabolic shift involving lipid and xenobiotic processes which, alongside the suppression of immune and structural programs, are changes that may be characteristic of early diploid transitional meningiomas.

Upregulation of xenobiotic-related and epoxygenase enzymes, including CYP2A6 and CYP2B6, may suggest an enhanced intrinsic capacity for lipid, steroid and oxidative metabolism. Rather than indicating drug resistance, which is not relevant in grade 1 meningiomas lacking systemic antitumoral therapy [75], this metabolic activity may support tumour homeostasis, redox balance and adaptation to microenvironmental stress [74]. The enrichment of ‘arachidonate epoxygenase activity’ further points to engagement of the arachidonic acid pathway, with epoxygenase products such as epoxyeicosatrienoic acids (EETs) having been implicated in angiogenesis and inflammatory modulation in other tumour-related contexts.

Together, these findings suggest that the diploid transitional WHO grade 1 meningioma subtype may depend on redox homeostasis and lipid-derived signalling. In this subtype, additional targeted studies would be required to evaluate whether these metabolic features represent exploitable vulnerabilities, for example through modulation of CYP-related pathways or arachidonic acid metabolism. This putative transcriptional pattern in DT-G1 meningiomas, characterized by attenuated immune and structural activity together with an oxidative-metabolic shift, would therefore reflect a molecular landscape that is distinct from that described in broader grade 1 cohorts [20,21].

#### 4.2.2. KEGG Pathway Enrichment Analysis

Dai et al. (2017) reported enrichment of proliferative and structural pathways such as ‘ECM–receptor interaction’, ‘PI3K–Akt’ and ‘cell adhesion’ in grade 1–3 meningiomas [20]. In contrast, our analysis of pure DT-G1 tumours suggests a transcriptional profile characterized by inflammatory attenuation and metabolic rewiring rather than proliferative signalling. Although both comparisons identified immune-related pathways (‘IL-17 signaling’, ‘TNF signaling’), we observed marked downregulation of key cytokines (IL6, CCL2, SELE, S100A8) consistent with reduced inflammatory activity and an “immune-cold-like” microenvironment. This aligns with reports of limited immune activation in grade 1 meningiomas [76] and might be compatible with diminished Th17-mediated inflammation (IL-17 axis) together with reduced TNF-dependent apoptotic and inflammatory cues. Together, these features that may facilitate tumour cell persistence within a permissive niche [72,73].

Enrichment of the cytoskeleton in muscle cell pathways may add a structural dimension to this immune attenuation. Downregulation of THBS1, MYH11, ATP1A2 and DCN, alongside upregulation of TNNC1, is consistent with altered contractile and cytoskeletal programs that may affect cell–matrix interactions, mechanical stability and tissue rigidity, consistent with disrupted ECM organization in grade 1 [74].

In our cohort, enrichments such as ‘arachidonic acid metabolism’, ‘drug metabolism–cytochrome P450’, ‘retinol metabolism’ and ‘chemical carcinogenesis–DNA adducts’, are indicative of metabolic and xenobiotic pathways not detected or emphasised in previous grade 1 studies [20,21], supporting a transcriptional signature characteristic of DT-G1 tumours. Upregulation of CYP2A6 and CYP2B6 may suggest enhanced processing of endogenous lipids, steroid derivatives and oxidative substrates, rather than drug-resistance mechanisms, which are not clinically relevant in grade 1 lesions [75]. Enrichment of epoxygenase-related activity within the arachidonic-acid cascade may promote EET production, with potential effects on angiogenesis and inflammatory modulation.

The enrichment of ‘chemical carcinogenesis—DNA adducts’ may reflect the involvement of xenobiotic and electrophile-processing pathways, consistent with oxidative DNA-damage responses reported in other CNS tumours [77]. Although nitrosamine-derived adducts have not been characterized in meningiomas, their inclusion within this KEGG category is compatible with a role of redox balance and detoxification metabolism even in early grade 1 contexts [78,79].

Finally, enrichment of the ‘Malaria’ pathway—previously reported in grade 1 comparisons [21]—may reflect endothelial activation and cytokine-linked innate immune modules, supporting the interplay between vascular and immune processes in low-grade tumours.

Collectively, these findings support a lipid/xenobiotic-oriented transcriptional landscape in our meningioma subtype, contrasting with proliferative and immunogenic programs reported when higher-grade tumours are analysed in pooled cohorts. This underscores the importance of stratifying analyses by histology and ploidy, as metabolic pathways such as ‘drug metabolism-cytochrome P450’, ‘arachidonic acid metabolism’ and ‘retinol metabolism’ may represent underexplored metabolic features in grade 1 biology.

#### 4.2.3. Hub Genes and PPI Network Interpretation

The PPI network (37 nodes and 180 edges) highlighted four highly connected hub genes (ASPN, SELE, ABCB1 and ACKR1) that are associated with extracellular matrix regulation, endothelial–immune modulation and xenobiotic metabolism, consistent with the inflammatory metabolic signature highlighted in our GO and KEGG analyses.

ASPN, the top-degree hub, is a matrix-associated protein associated with collagen binding and ECM organization, and its downregulation is consistent with the reduced ECM organization observed in our GO analysis, suggesting altered matrix architecture and stromal interactions in DT-G1 meningiomas [80]. SELE (E-selectin), an endothelial adhesion molecule induced by inflammatory cytokines, is associated with vascular and immune modules, and its suppression parallels the downregulated TNF and IL-17 pathways, consistent with reduced endothelial activation and weakened leukocyte–endothelium communication [81]. ACKR1 (DARC), an atypical chemokine-scavenging receptor, is associated with the reduced expression of IL6, CCL2 and other chemokines, consistent with altered chemokine handling and limited immune-cell trafficking in this subset [82]. ABCB1, an ATP-dependent efflux transporter, is associated with xenobiotic transport with metabolic stress responses, and its upregulation is consistent with the enrichment of xenobiotic-related pathways, likely reflecting detoxification and redox adaptation rather than clinically relevant drug-resistance mechanisms in DT-G1 meningiomas [83,84].

Taken together, the combined downregulation of ASPN, SELE and ACKR1, alongside the upregulation of ABCB1, supports a hub-level transcriptional signature characteristic of DT-G1 meningiomas. This pattern is associated with ECM remodelling, endothelial–immune modulation and xenobiotic handling, and is compatible with an immune-tolerant, metabolically adapted tumour microenvironment thereby providing an integrative framework for future investigation.

Notably, within the framework of an exploratory analysis, the observed transcriptomic changes appear biologically coherent and align with pathways commonly associated with cancer-related processes and meningioma biology.

### 4.3. Integrative Summary

Taken together, our findings support the view that DT-G1 meningiomas display a coherent inflammatory–metabolic programme, characterized by attenuated immune signalling and pronounced lipid/xenobiotic metabolic activity. This pattern is consistent with the immunocytochemical phenotype observed in our cultures. The hub genes ASPN, SELE, ACKR1 and ABCB1 are associated with key components of these processes: ASPN with extracellular matrix remodelling, SELE and ACKR1 with endothelial–immune modulation, and ABCB1 with xenobiotic transport and metabolic stress adaptation. Collectively, this hub-level pattern is compatible with a distinct metabolic–immune phenotype in DT-G1 meningiomas and offers an integrative framework that would link immune attenuation, matrix dynamics and lipid/xenobiotic metabolism.

Importantly, this transcriptomic landscape is consistent with the immunocytochemical profile characterized by CB1/CB2, vimentin, α-SMA, nestin and Cx43 expression. This consistency supports the interpretation that the inflammatory metabolic and cytoskeletal programs identified likely reflect features of DT-G1 meningiomas rather than artefacts of culture conditions.

Overall, this integrated molecular profile supports the relevance of stratifying meningiomas by histology and ploidy to uncover biologically meaningful subgroups. It points to metabolic pathways and their regulatory networks as relevant avenues for further mechanistic exploration in grade 1 meningiomas.

A limitation of this study is the relatively small number of samples in the selected subset of the Tabernero et al. dataset [10]. In this context, exploratory analysis of raw expression values (PCA and hierarchical clustering) showed a clear separation between normal meninges and meningiomas, consistent with biological robustness of the subdataset, while acknowledging that subtle batch effects related to the use of publicly available microarray data cannot be entirely excluded. In this regard, the empirical Bayes statistics implemented in the *limma* framework stabilize variance estimates when sample size is limited. Even so, additional independent cohorts would further strengthen the generalizability of these findings. Although H16T-MG did not undergo cytogenetic analysis, this does not affect our conclusions, as it was used exclusively for immunocytochemical and methodological support.

In addition, the transcriptomic analysis was conceived as exploratory by design and should be interpreted as providing biological context and hypothesis-generating insights rather than definitive molecular classification. Accordingly, the transcriptomic findings are intended to support and contextualize the in vitro cellular observations rather than to drive standalone molecular conclusions.

Another limitation of this study is the inability to retrospectively test the WHO 2021 molecular grade-defining markers (TERT-promoter mutation and homozygous CDKN2A/B deletion) [34,35,36] However, multiple clinico-cytogenetic features, strict diploidy, and >5-year recurrence-free clinical course render these alterations unlikely, as supported by large cytogenetic and multi-institutional cohorts [10,33,37,38,40,71]. Taken together, this evidence supports the interpretation of our tumours within a WHO 2021 grade 1 biological framework.

## 5. Conclusions

This study reports the first integrated transcriptomic and in vitro characterization of diploid transitional WHO grade I meningiomas. By establishing two long-term, genetically unmanipulated grade 1 meningioma cultures and performing contextual analysis of a subtype-restricted microarray dataset, we describe a reproducible inflammatory–metabolic signature characterized by attenuated cytokine signalling, low-remodelling ECM programs and pronounced lipid/xenobiotic metabolic activity.

Hub-gene analysis (ASPN, SELE, ACKR1, ABCB1) further supports the presence of coordinated alterations in matrix organisation, endothelial–immune communication and detoxification pathways, consistent with a distinct microenvironmental and metabolic profile for this subtype. Importantly, the exploratory transcriptomic patterns were consistent with immunocytochemical phenotype observed in our cultures, supporting the biological relevance in vitro models within a descriptive model-establishment framework for study of DT-G1 meningiomas.

Taken together, these findings appear to support the relevance of histological and ploidy-based stratification in meningioma research and point to metabolic pathways—particularly lipid and xenobiotic metabolism—as biological features warranting further mechanistic investigation in WHO grade 1 meningiomas.

## Figures and Tables

**Table 1:** Differentially expressed genes (DEG) identified in DT-G1 meningiomas compared with healthy meningeal tissue.

ProbeID	Gene Symbol	Gene Name	log2FC	Direction
201131_s_at	ABCB1	ATP-binding cassette sub-family B member 1	3.115135	↑
209994_s_at	ABCB1	ATP-binding cassette sub-family B member 1	3.946437	↑
201596_x_at	KRT18	Keratin 18	3.145330	↑
219529_at	CLIC3	Chloride intracellular channel 3	3.886168	↑
216050_at	CYP2A6	Cytochrome P450 family 2 subfamily A member 6	3.574162	↑
209994_s_at	CYP2B6	Cytochrome P450 family 2 subfamily B member 6	4.209101	↑
201123_s_at	EIF5A	Eukaryotic translation initiation factor 5A	3.191908	↑
204875_s_at	GMDS	GDP-mannose 4,6-dehydratase	3.065947	↑
204987_at	ITIH2	Inter-alpha-trypsin inhibitor heavy chain 2	3.116748	↑
221583_at	KCNMA1	Potassium calcium-activated channel subfamily M alpha 1	3.593002	↑
201131_s_at	CDH1	Cadherin 1	3.115135	↑
215894_at	PTGDR	Prostaglandin D2 receptor	3.793593	↑
209904_at	TNNC1	Troponin C1, slow skeletal and cardiac type	4.498355	↑
202237_at	NNMT	Nicotinamide N-methyltransferase	−4.718362	↓
202238_s_at	NNMT	Nicotinamide N-methyltransferase	−4.040629	↓
209335_at	DCN	Decorin	−4.344368	↓
201893_x_at	DCN	Decorin	−3.832883	↓
211813_x_at	DCN	Decorin	−3.835581	↓
208335_s_at	ACKR1	Atypical chemokine receptor 1 (Duffy blood group)	−3.031395	↓
221031_s_at	APOLD1	Apolipoprotein L domain containing 1	−3.898835	↓
222162_s_at	ADAMTS1	ADAM metallopeptidase with thrombospondin type 1 motif 1	−4.334914	↓
217546_at	ADAMTS1	ADAM metallopeptidase with thrombospondin type 1 motif 1	−4.334914	↓
2000897_s_at	PALLD	Palladin, cytoskeletal associated protein	−4.227503	↓
200907_s_at	PALLD	Palladin, cytoskeletal associated protein	−3.904815	↓
202672_s_at	ATF3	Activating transcription factor 3	−3.336215	↓
221541_at	CRISPLD2	Cysteine-rich secretory protein LCCL domain containing 2	−3.089719	↓
217546_at	MT1M	Metallothionein 1M	−3.647168	↓
201645_at	TNC	Tenascin C	−3.478160	↓
205207_at	IL6	Interleukin 6	−3.655819	↓
209774_x_at	CXCL2	C-X-C motif chemokine ligand 2	−4.770032	↓
204288s_at	SORBS2	Sorbin and SH3 domain containing 2	−3.122188	↓
36711_at	MAFF	MAF bZIP transcription factor F	−4.765884	↓
207961_x_at	MYH11	Myosin heavy chain 11	−3.146901	↓
201496_x_at	MYH11	Myosin heavy chain 11	−4.208234	↓
201497_x_at	MYH11	Myosin heavy chain 11	−4.147030	↓
203296_s_at	ATP1A2	ATPase Na+/K+ transporting subunit alpha 2	−3.426334	↓
216834_at	RGS1	Regulator of G protein signalling 1	−3.224255	↓
214677_x_at	KDM2A	Lysine demethylase 2A	−3.169647	↓
211430_s_at	PDCD1	Programmed cell death 1	−3.879132	↓
217022_s_at	HBB	Haemoglobin subunit beta	−3.481622	↓
209116_x_at	HBB	Haemoglobin subunit beta	−3.118670	↓
203485_at	RTN1	Reticulon 1	−3.904815	↓
212865_s_at	COL14A1	Collagen type XIV alpha 1 chain	−3.347500	↓
209470_s_at	GPM6A	Glycoprotein M6A	−3.270120	↓
209469_at	GPM6A	Glycoprotein M6A	−4.030587	↓
209686_at	S100B	S100 calcium-binding protein B	−3.012154	↓
202508_s_at	SNAP25	Synaptosome associated protein 25	−3.511881	↓
201109_s_at	THBS1	Thrombospondin 1	−3.238524	↓
201110_s_at	THBS1	Thrombospondin 1	−3.515628	↓
204748_at	PTGS2	Prostaglandin-endoperoxide synthase 2	−3.297718	↓
206211_at	SELE	Selectin E	−3.334817	↓
210198_s_at	PLP1	Proteolipid protein 1	−4.037846	↓
219087_at	ASPN	Asporin	−3.669058	↓
209335_at	MBP	Myelin basic protein	−5.808051	↓
204939_s_at	PLN	Phospholamban	−4.402870	↓
202917_s_at	S100A8	S100 calcium-binding protein A8	−3.948918	↓
203540_at	GFAP	Glial fibrillary acidic protein	−3.290292	↓
221651_x_at	CYP2C19	Cytochrome P450 family 2 subfamily C member 19	−3.067765	↓
221671_x_at	CYP2C9	Cytochrome P450 family 2 subfamily C member 9	−3.070996	↓
216598_s_at	CCL2	C-C motif chemokine ligand 2	−3.269667	↓
200974_at	ACTA2	Actin alpha 2, smooth muscle	−3.409686	↓

Notes: Probe identifiers, gene symbols, standardized gene names, log_2_ fold change (log_2_FC), and direction of deregulation relative to normal meninges are shown. Upward arrows (↑) indicate upregulation in meningioma samples, whereas downward arrows (↓) indicate downregulation. Gene names were standardized to English biomedical nomenclature, and symbol corrections were applied when necessary (e.g., *GPM6A*).

**Table 4:** Comparative analysis of GO enrichment, KEGG pathways, and hub genes across three studies using GSE43290 dataset.

Study	Key GO Terms	Key KEGG Pathways	Hub Genes (PPI)
**Dai et al., 2017 [20]** WHO grades 1, 2 + 3 vs. Meninges	*Extracellular matrix organization* *Cell adhesion* Angiogenesis Signal transduction	AGE-RAGE signalling pathway in diabetic complications PI3K-Akt signalling pathway **TNF signalling pathway** ECM-receptor interaction; Focal adhesion **IL-17 signalling pathway** MAPK signalling pathway NF-κB signalling pathway	JUN PIK3R1 FOS AGT MYC
**Cao et al., 2020 [21]** WHO grade 1 (all types) vs. Meninges	*Extracellular matrix organization* **Inflammatory response** *Cell adhesion* **Extracellular space** Integrin binding	*Malaria* Focal adhesion	AGT CXCL8 CXCL2 CXCL12 CXCR4
**Current Study** **DT-G1** vs. **Meninges**	**Inflammatory response** “Arachidonate epoxygenase activity” “Epoxygenase P450 pathway” “Xenobiotic catabolic process” Collagen-containing extracellular matrix Extracellular region **Extracellular space** Heme binding Monooxygenase activity “Oxidoreductase activity” *Extracellular matrix organization* (low significance) *Cell adhesion* (low significance)	“Lipid and aterosclerosis” *Malaria* **IL-17 signalling pathway** **TNF signalling pathway** “Cytoskeleton in muscle cells” “Arachidonic acid metabolism” “Chemical carcinogenesis—DNA adducts” “Drug metabolism—cytochrome P450” “African tripanosomiasis” “Retinol metabolism”	ASPN SELE ACKR1 ABCB1

Notes: Bold indicates GO terms or KEGG pathways shared with at least one previous study [20,21]. Italics denote terms common to all three studies and quotation marks (“ ”) indicate terms unique to the present study.

## Data Availability

The microarray data used in the bioinformatic analyses were obtained from the publicly available dataset published by Tabernero et al. [20] and downloaded from the Gene Expression Omnibus (GEO) repository (accession number: GSE43290). No new genomic or clinical datasets were generated for this study. The H8T-MG and H16T-MG cell lines were derived from historical anonymized surgical material and are not publicly available due to ethical and institutional restrictions.

## Supplementary Materials

The following supporting information can be downloaded at https://www.mdpi.com/article/10.3390/biom16050744/s1, Table S1: Primary antibodies used in this study. Table S2: Secondary antibodies used in this study. Table S3: GEO accession numbers, biological sample descriptions, and internal codes assigned to the Tabernero dataset. Table S4: Full editable KEGG pathway enrichment table corresponding to Table 3.
