# Supplementary material for: Establishment of the H8T-MG Meningioma Cell Line and Integrated Transcriptomics Reveal a Metabolic–Immune Signature in Diploid Transitional WHO Grade 1 Tumours
Source: Biomolecules. 2026 May 19;16(5):744. doi: 10.3390/biom16050744 (PMC13204870; doi:10.3390/biom16050744)
Supplement: Supplementary file 1 [file biomolecules-16-00744-s001.zip › Supplementary Table S2.pdf]

Table S2. Secundary antibodies used in this study

Secondary antibody	Host species	Dilution	Supplier
Alexa Fluor 488 goat anti-mouse IgG (H+L)	Goat anti-mouse	1:500	Molecular Probes Europe BV
Alexa Fluor 488 goat anti-rabbit IgG (H+L)	Goat anti-rabbit	1:200	Molecular Probes Europe BV
Alexa Fluor 546 goat anti-mouse IgG (H+L)	Goat anti-mouse	1:500	Molecular Probes Europe BV
Alexa Fluor 568 goat anti-rabbit IgG (H+L)	Goat anti-rabbit	1:500	Molecular Probes Europe BV
Alexa Fluor 568 donkey anti-goat IgG (H+L)	Donkey anti-goat	1:500	Molecular Probes Europe BV
Alexa Fluor 546 goat anti-mouse IgG (H+L)	Goat anti-mouse	1:300	Molecular Probes Europe BV
Biotinylated anti-goat IgG (H+L)	Rabbit anti-goat	1:100	Vector Laboratories
Biotinylated anti-mouse IgG (H+L)	Goat anti-mouse	1:100	Vector Laboratories
Biotinylated anti-rabbit IgG (H+L)	Goat anti-rabbit	1:100	Vector Laboratories
Cy2-conjugated streptavidin	—	1:500	Amersham
Cy3-conjugated streptavidin	—	1:1000	Amersham
