# Supplementary material for: Establishment of the H8T-MG Meningioma Cell Line and Integrated Transcriptomics Reveal a Metabolic–Immune Signature in Diploid Transitional WHO Grade 1 Tumours
Source: Biomolecules. 2026 May 19;16(5):744. doi: 10.3390/biom16050744 (PMC13204870; doi:10.3390/biom16050744)
Supplement: Supplementary file 1 [file biomolecules-16-00744-s001.zip › Supplementary Table S4.pdf]

Table S4. GO Enrichment Analysis of DEGs in Grade 1 Transitional Diploid Meningioma vs. Healthy Meninges

Category	GO Term	% Genes	P-value	Adjusted P-value (Benjamini)
GOTERM_BP_DIRECT	inflammatory response	18%	9,52E-06	0,00474
GOTERM_MF_DIRECT	arachidonate epoxygenase activity	8%	1,03E-05	0,00194
GOTERM_BP_DIRECT	epoxygenase P450 pathway	8%	1,55E-05	0,00474
GOTERM_CC_DIRECT	collagen-containing extracellular matrix	16%	3,21E-05	0,00517
GOTERM_BP_DIRECT	xenobiotic catabolic process	8%	3,51E-05	0,00714
GOTERM_MF_DIRECT	heme binding	12%	4,68E-05	0,00443
GOTERM_MF_DIRECT	oxidoreductase activity, acting on paired donors, with incorporation or reduction of molecular oxygen, reduced flavin or flavoprotein as one donor, and incorporation of one atom of oxygen	8%	0,000104	0,00657
GOTERM_CC_DIRECT	extracellular matrix	12%	0,000176	0,01
GOTERM_CC_DIRECT	extracellular region	32%	0,000186	0,01
GOTERM_BP_DIRECT	xenobiotic metabolic process	10%	0,00023	0,0348
GOTERM_CC_DIRECT	extracellular space	28%	0,000284	0,0115
GOTERM_BP_DIRECT	steroid metabolic process	8%	0,000285	0,0348
GOTERM_MF_DIRECT	oxidoreductase activity, acting on paired donors, with incorporation or reduction of molecular oxygen	8%	0,000456	0,0215
GOTERM_CC_DIRECT	caveola	8%	0,000737	0,0237
GOTERM_BP_DIRECT	astrocyte development	6%	0,00079	0,0803
GOTERM_MF_DIRECT	monooxygenase activity	8%	0,000884	0,0334
GOTERM_CC_DIRECT	endoplasmic reticulum lumen	12%	0,000891	0,0239
GOTERM_BP_DIRECT	regulation of blood pressure	8%	0,000939	0,0818
GOTERM_CC_DIRECT	lamellipodium	10%	0,00106	0,0243
GOTERM_MF_DIRECT	calcium-dependent protein binding	8%	0,00119	0,0375
GOTERM_BP_DIRECT	long-chain fatty acid biosynthetic process	6%	0,00121	0,0924
GOTERM_CC_DIRECT	neuronal cell body	12%	0,00172	0,0346
GOTERM_BP_DIRECT	maintenance of blood-brain barrier	6%	0,00317	0,215
GOTERM_BP_DIRECT	response to xenobiotic stimulus	10%	0,00355	0,217
GOTERM_CC_DIRECT	actin cytoskeleton	10%	0,00485	0,0868
GOTERM_MF_DIRECT	(R)-limonene 6-monooxygenase activity	4%	0,00508	0,107
GOTERM_MF_DIRECT	(S)-limonene 6-monooxygenase activity	4%	0,00508	0,107
GOTERM_MF_DIRECT	(S)-limonene 7-monooxygenase activity	4%	0,00508	0,107
GOTERM_MF_DIRECT	iron ion binding	8%	0,00598	0,113
GOTERM_BP_DIRECT	positive regulation of gene expression	12%	0,00688	0,368
GOTERM_BP_DIRECT	extracellular matrix organization	8%	0,00768	0,368
GOTERM_BP_DIRECT	positive regulation of smooth muscle cell proliferation	6%	0,00784	0,368
GOTERM_BP_DIRECT	immune response	12%	0,0101	0,429
GOTERM_BP_DIRECT	humoral immune response	6%	0,0106	0,429
GOTERM_BP_DIRECT	cellular response to lipopolysaccharide	8%	0,0118	0,437
GOTERM_BP_DIRECT	cellular response to prostaglandin D stimulus	4%	0,0123	0,437
GOTERM_BP_DIRECT	chemokine-mediated signaling pathway	6%	0,0129	0,437
GOTERM_CC_DIRECT	extracellular exosome	24%	0,0136	0,199
GOTERM_BP_DIRECT	axon ensheathment	4%	0,0147	0,448
GOTERM_BP_DIRECT	negative regulation of heart contraction	4%	0,0147	0,448
GOTERM_BP_DIRECT	monoterpenoid metabolic process	4%	0,0171	0,497
GOTERM_BP_DIRECT	neurotransmitter uptake	4%	0,0195	0,542
GOTERM_CC_DIRECT	endoplasmic reticulum membrane	16%	0,0204	0,272

GOTERM_CC_DIRECT	basement membrane	6%	0,022	0,272
GOTERM_MF_DIRECT	estrogen 2-hydroxylase activity	4%	0,0227	0,39
GOTERM_BP_DIRECT	regulation of neuroinflammatory response	4%	0,0244	0,605
GOTERM_BP_DIRECT	chronic inflammatory response	4%	0,0244	0,605
GOTERM_BP_DIRECT	regulation of inflammatory response	6%	0,0257	0,605
GOTERM_BP_DIRECT	organic acid metabolic process	4%	0,0268	0,605
GOTERM_BP_DIRECT	negative regulation of calcium ion transport	4%	0,0268	0,605
GOTERM_MF_DIRECT	RAGE receptor binding	4%	0,0276	0,402
GOTERM_MF_DIRECT	structural constituent of myelin sheath	4%	0,0276	0,402
GOTERM_CC_DIRECT	interstitial matrix	4%	0,0279	0,321
GOTERM_BP_DIRECT	omega-hydroxylase P450 pathway	4%	0,0292	0,633
GOTERM_BP_DIRECT	regulation of muscle contraction	4%	0,0316	0,633
GOTERM_CC_DIRECT	intracellular membrane-bounded organelle	14%	0,0321	0,344
GOTERM_MF_DIRECT	identical protein binding	20%	0,0334	0,416
GOTERM_BP_DIRECT	regulation of cardiac muscle cell contraction	4%	0,034	0,633
GOTERM_BP_DIRECT	relaxation of cardiac muscle	4%	0,034	0,633
GOTERM_BP_DIRECT	leukocyte migration involved in inflammatory response	4%	0,034	0,633
GOTERM_BP_DIRECT	Notch signaling pathway	6%	0,0343	0,633
GOTERM_BP_DIRECT	positive regulation of cell population proliferation	10%	0,037	0,663
GOTERM_MF_DIRECT	steroid hydroxylase activity	4%	0,0375	0,416
GOTERM_MF_DIRECT	extracellular matrix structural constituent	6%	0,038	0,416
GOTERM_MF_DIRECT	calcium ion binding	12%	0,039	0,416
GOTERM_MF_DIRECT	extracellular matrix structural constituent conferring compression resistance	4%	0,04	0,416
GOTERM_BP_DIRECT	intracellular potassium ion homeostasis	4%	0,0411	0,696
GOTERM_BP_DIRECT	regulation of the force of heart contraction	4%	0,0411	0,696
GOTERM_MF_DIRECT	protein homodimerization activity	12%	0,0418	0,416
GOTERM_BP_DIRECT	negative regulation of angiogenesis	6%	0,0431	0,711
GOTERM_BP_DIRECT	cell adhesion	10%	0,0484	0,758
GOTERM_BP_DIRECT	potassium ion transmembrane transport	6%	0,0485	0,758
GOTERM_BP_DIRECT	positive regulation of macrophage chemotaxis	4%	0,0529	0,806
GOTERM_BP_DIRECT	cytoskeleton organization	6%	0,0566	0,816
GOTERM_BP_DIRECT	regulation of cardiac muscle contraction by regulation of the release of sequestered calcium ion	4%	0,0575	0,816
GOTERM_BP_DIRECT	negative regulation of endothelial cell migration	4%	0,0575	0,816
GOTERM_BP_DIRECT	monocyte chemotaxis	4%	0,0599	0,83
GOTERM_MF_DIRECT	glycosaminoglycan binding	4%	0,0618	0,564
GOTERM_MF_DIRECT	integrin binding	6%	0,0626	0,564
GOTERM_CC_DIRECT	myelin sheath	4%	0,0684	0,688
GOTERM_BP_DIRECT	positive regulation of endothelial cell apoptotic process	4%	0,0691	0,937
GOTERM_BP_DIRECT	response to tumor necrosis factor	4%	0,0737	0,977
GOTERM_BP_DIRECT	positive regulation of vascular endothelial growth factor production	4%	0,076	0,986
GOTERM_MF_DIRECT	heparin binding	6%	0,0761	0,654
GOTERM_BP_DIRECT	positive regulation of synaptic transmission, glutamatergic	4%	0,0783	0,995
GOTERM_BP_DIRECT	cell-cell adhesion	6%	0,0841	1.00e+0
GOTERM_MF_DIRECT	peroxidase activity	4%	0,0877	0,717

GOTERM_BP_DIRECT	negative regulation of cell population proliferation	8%	0,0907	1.00e+0
GOTERM_MF_DIRECT	oxygen binding	4%	0,0924	0,717
GOTERM_CC_DIRECT	cell projection	6%	0,0963	0,861

Notes:
Gene Ontology (GO) enrichment analysis of differentially expressed genes (DEGs) in grade 1 transitional diploid meningioma compared to healthy meninges. Over-represented GO terms are categorized by Biological Process (BP), Molecular Function (MF), and Cellular Component (CC), with corresponding percentages of associated genes, raw p-values, and Benjamini-adjusted p-values indicating statistical significance.
