# Supplementary material for: Establishment of the H8T-MG Meningioma Cell Line and Integrated Transcriptomics Reveal a Metabolic–Immune Signature in Diploid Transitional WHO Grade 1 Tumours
Source: Biomolecules. 2026 May 19;16(5):744. doi: 10.3390/biom16050744 (PMC13204870; doi:10.3390/biom16050744)
Supplement: Supplementary file 1 [file biomolecules-16-00744-s001.zip › Supplementary Table S1.pdf]

Table S1. Primary antibodies used in this study

Antigen	Clone	Host species	Dilution	Supplier
alpha-SMA	Clone 1A4	Mouse	*1:100*	InmunoStar
CB1	Polyclonal	Rabbit	*1:750*	Affinity Bioreagents
CB2	Polyclonal	Rabbit	*1:500*	Sigma
Conexin-43	Polyclonal	Rabbit	*1:500*	Sigma
Nestin	Polyclonal	Rabbit	*1:1000*	Chemicon (Temecula, CA, USA)
NF-L	Polyclonal	Rabbit	*1:600*	AbD Serotec (Oxford, UK)
NF-M*	Clone M14	Mouse	*1:10*	Gift from Dr. Luque (Instituto de Neurociencias, CSIC-UMH, Alicante, Spain)
NF-M	Clone M20	Mouse	*1:10*	Gift from Dr. Luque (Instituto de Neurociencias, CSIC-UMH, Alicante, Spain; after Hornung & Riederer, 1999, [19])
NF-M	Clone M20	Mouse	*1:10*	Gift from Dr. Luque (Instituto de Neurociencias, CSIC-UMH, Alicante, Spain; after Hornung & Riederer, 1999, [19])
NF-160	n/a	Mouse	*1:300*	Sigma
RC2	RC2	Mouse	*1:100*	Hybridoma Bank (DSHB, Iowa City, USA)
Vimentin	Clone V9	Mouse	*1:50*	Sigma

* This antibody corresponds to an NF-M monoclonal antibody (Clone M14) provided by Dr. Luque (Instituto de Neurociencias, CSIC-UMH, Alicante, Spain) No original published reference for this antibody could be verified.
