# Supplementary material for: Establishment of the H8T-MG Meningioma Cell Line and Integrated Transcriptomics Reveal a Metabolic–Immune Signature in Diploid Transitional WHO Grade 1 Tumours
Source: Biomolecules. 2026 May 19;16(5):744. doi: 10.3390/biom16050744 (PMC13204870; doi:10.3390/biom16050744)
Supplement: Supplementary file 1 [file biomolecules-16-00744-s001.zip › Supplementary Table S3.pdf]

Table S3. GEO accession numbers, biological sample descriptions, and internal codes assigned to the Tabernero dataset.

GEO Accession	Biological Sample Description	Internal Code
GSM 1060112	Sample 1 normal meninges	NormalM1
GSM 1060113	Sample 2 normal meninges	NormalM2
GSM 1060114	Sample 3 normal meninges	NormalM3
GSM 1060115	Sample 4 normal meninges	NormalM4
GSM 1060067	Meningioma #3 tumor	D3
GSM 1060068	Meningioma #4 tumor	D4
GSM 1060069	Meningioma #5 tumor	D5
GSM 1060070	Meningioma #6tumor	D6
GSM 1060073	Meningioma #9tumor	D10
GSM 1060074	Meningioma #10tumor	D11
GSM 1060077	Meningioma #13tumor	D14
GSM 1060078	Meningioma #14tumor	D15
